# Case Report: Neuromyelitis Optica Spectrum Disorder With Progressive Elevation of Cerebrospinal Fluid Cell Count and Protein Level Mimicking Infectious Meningomyelitis: A Diagnostic Challenge

**DOI:** 10.3389/fimmu.2022.864664

**Published:** 2022-05-19

**Authors:** Yin-Xi Zhang, Meng-Ting Cai, Ming-Xia He, Yu-Qiang Lu, Xiao Luo, Tian-Yi Zhang

**Affiliations:** ^1^ Department of Neurology, Second Affiliated Hospital, School of Medicine, Zhejiang University, Hangzhou, China; ^2^ Department of Hematology, Tongde Hospital of Zhejiang Province, Hangzhou, China; ^3^ Department of Neurology, Tongde Hospital of Zhejiang Province, Hangzhou, China; ^4^ Department of Radiology, Second Affiliated Hospital, School of Medicine, Zhejiang University, Hangzhou, China

**Keywords:** neuromyelitis optica spectrum disorder, aquaporin-4 antibody, infectious meningomyelitis, cerebrospinal fluid, longitudinally extensive transverse myelitis, area postrema syndrome

## Abstract

Neuromyelitis optica spectrum disorder (NMOSD) is an autoimmune-mediated idiopathic inflammatory demyelinating disease with a typical clinical presentation of optic neuritis, acute myelitis, and area postrema syndrome. Most NMOSD patients are seropositive for disease-specific and pathogenic aquaporin-4 (AQP4) antibodies, which are key markers for the NMOSD diagnosis. Herein, we report an atypical case of a 41-year-old man who complained of intractable hiccups and vomiting at disease onset, followed by fever, headache, back pain, progressive paresthesia, and weakness of extremities later on. Magnetic resonance imaging revealed longitudinally extensive transverse myelitis. Cerebrospinal fluid analysis showed progressive increases in the white blood cell count and the protein level, which were accompanied by the deterioration of clinical manifestations. The patient was initially suspected of infectious meningomyelitis but was finally diagnosed with NMOSD. This case with distinct cerebrospinal fluid findings broadens the phenotypic spectrum of NMOSD. Furthermore, it also highlights the clinical value of AQP4 antibody test for early definitive diagnosis and proper treatment.

## Introduction

Neuromyelitis optica (NMO) is an autoimmune-mediated idiopathic inflammatory demyelinating disease that predominantly affects the optic nerve and spinal cord. The 2015 International Panel for NMO Diagnosis introduced neuromyelitis optica spectrum disorder (NMOSD) to include a broader spectrum and established its diagnostic criteria (1). NMOSD is characterized by recurrent attacks of optic neuritis, transverse myelitis, and area postrema syndrome (APS). Typical neuroradiological findings include long-segment involvement of the optic nerve (particularly when simultaneous bilateral and extending posteriorly into the optic chiasm), longitudinally extensive transverse myelitis (LETM), and lesions of the dorsal medulla (especially the area postrema) (2). The majority of patients with NMOSD are seropositive for disease-specific and pathogenic aquaporin-4 (AQP4) antibodies, which are key markers for its diagnosis (1, 3). The wide application of AQP4 antibody test expands the phenotype of uncommon clinical manifestations of this disease. Here, we present an atypical case of NMOSD with fever, headache, back pain, progressive paresthesia, weakness of the extremities, and continuously elevating white blood cell (WBC) count, and protein level in the cerebrospinal fluid (CSF). These misdirecting manifestations seemed to all lead to infectious meningomyelitis in this case; however, the patient was finally diagnosed with NMOSD.

## Case Description

**Figure 1:**
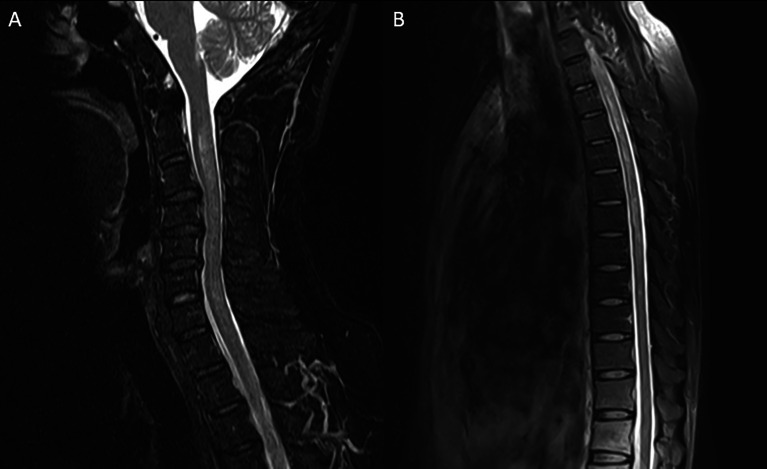
Magnetic resonance imaging of the cervical **(A)** and thoracic **(B)** spinal cord. Sagittal T2-weighted imaging showed longitudinally extensive hyperintense lesion extending from C2 to T8.

**Figure 2:**
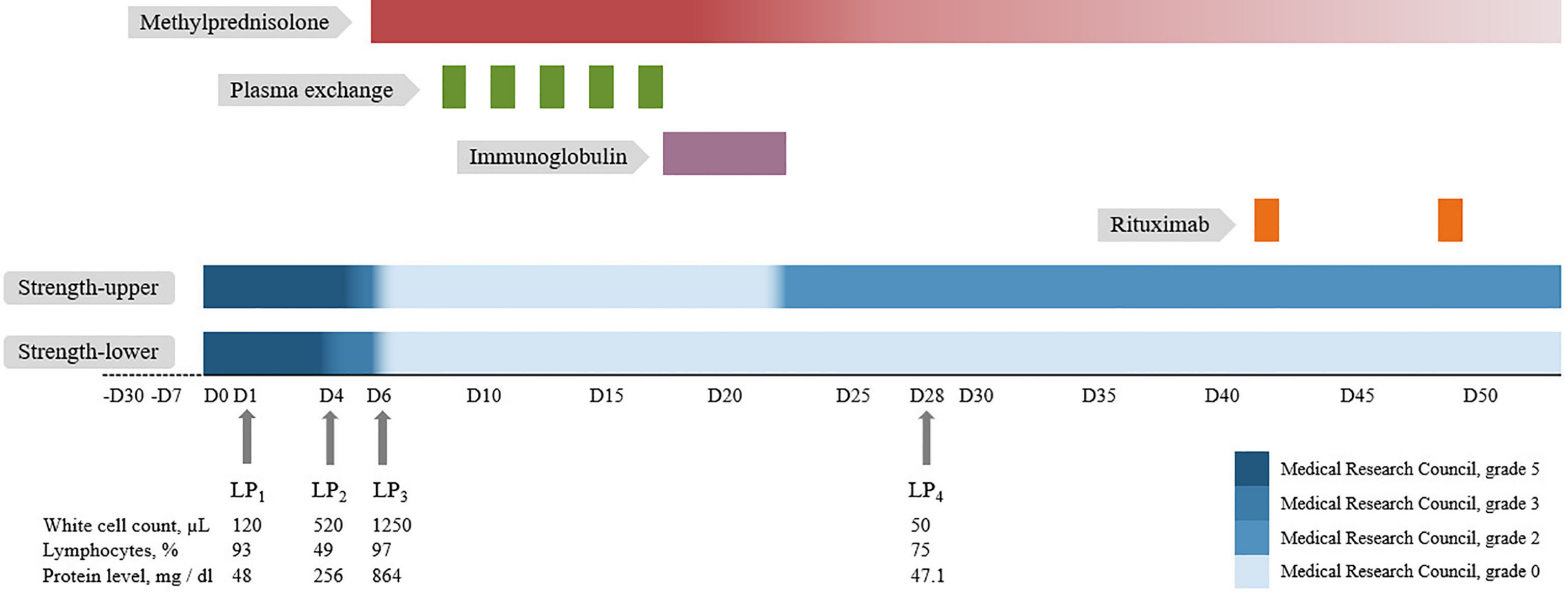
Timeline depicting muscle strength change, immunotherapy, and cerebrospinal fluid analysis of the patient. For further details, refer to the main text. LP, lumbar puncture.

A 41-year-old previously healthy man was sent to the hospital with a 1-month history of intractable hiccups and vomiting and a 1-week history of headache, back pain, and ascending paresthesia. Neurological examination on admission showed intact cranial nerves, neck rigidity, hypoesthesia below the T6 level, vibration hypoesthesia, bilateral positive Babinski signs, and normal muscle strength. Routine workup including complete blood cell count, basic metabolic panel, tumor markers, and rheumatology panel was normal. Chest computerized tomography (CT), abdominal ultrasound, and brain magnetic resonance imaging (MRI) were unremarkable. Spinal MRI demonstrated longitudinally extensive T2 hyperintensities from C2 to T8 without enhancement (**Figure 1**). Spinal contrast-enhanced MR angiography showed no abnormalities. Initial lumbar puncture revealed a CSF opening pressure of 140 mmH_2_O, a WBC count of 120/µl (93% lymphocytes), a protein level of 48 mg/dl, and a normal glucose level (**Figure 2**). No intrathecal oligoclonal bands were found.

Three days later, the patient developed progressive weakness of lower extremities (Medical Research Council, grade 3) with dysuria and constipation and fever up to 38°C, while all serum infectious indicators (e.g., leukocytes, procalcitonin test, C-reactive protein, and cultures), and urinalysis/urine culture were negative. Therefore, a reexamination of the lumbar puncture was performed, revealing an increased WBC count (520/µl) and protein level (255.5 mg/dl). CSF next-generation sequencing (NGS) for bacteria, viruses, fungi, and parasites with known genome sequences and cultures for bacteria and fungi were negative. Moreover, the CSF cytology was suspicious for tumor cells. Fluorodeoxyglucose positron emission tomography/CT showed increased metabolism in multiple segments of the cervical and thoracic regions and no evidence of systemic tumors.

In the following 2 days, the weakness progressed to all extremities (Medical Research Council, grade 0) and anesthesia arose to the C3 level, accompanied by hoarseness, dysphagia, dysuria, dyspnea, and a peak temperature of 38.8°C. The reexamination of serum and urine showed no evidence of infection. Based on the evaluation, neither tumor nor infection of the central nervous system could be ruled out. A third lumbar puncture was performed, which showed a WBC count of 1,250/µl (97% lymphocytes) and a protein level of 864.4 mg/dl. Flow cytometry and repeated NGS analysis in CSF were unrevealing. The results of the AQP4 antibody test at admission returned, demonstrating positivity in both the serum (titer, 1:100) and the CSF (titer, 1:3.2) using cell-based assay. The final diagnosis of NMOSD was made.

The patient was then treated with intravenous methylprednisolone (500 mg/day for 5 days, followed by a tapering scheme) combined with plasma exchange (5 cycles, every other day) and intravenous immunoglobulin (0.4 g/kg per day for 5 days) (**Figure 2**). After the first-line treatment, muscle strength of the upper extremities recovered to grade 2 and anesthesia decreased to the level of T4. The symptoms of hiccup and vomiting disappeared completely. A reexamination of the lumbar puncture revealed that the CSF WBC count and protein level decreased significantly. The reexamined serum AQP4 antibody titer was 1:10. Rituximab (500 mg, twice) was administered for sequential therapy. In the follow-up 3 months after discharge, the patient could lift both upper extremities but suffered from pain all over the body. The movement of the lower extremities and the tactile sensation below the xiphoid process were still poor.

## Discussion

Here, we report an NMOSD patient who successively presented with hiccups, vomiting, headache, back pain, fever, paresthesia, and weakness of the extremities. In addition, the CSF tests also showed progressive increases in the WBC count and the protein level consistently with the aggravation of myelitis symptoms, which has not been reported before. Although the patient had infectious meningomyelitis-like symptoms, the repeated tests showed no evidence of infection. Nevertheless, the involvement of infection was difficult to completely exclude, leading to a diagnostic challenge. However, the detection of AQP4 antibodies in both serum and CSF with the clinical manifestations of APS and acute myelitis, according to the 2015 diagnostic criteria, makes the definite diagnosis of NMOSD. Furthermore, the fact that the patient’s symptoms improved after immunotherapy, rather than anti-infective treatment, also supported this diagnosis.

The intractable hiccups and vomiting that the patient suffered initially were ignored at the time, while those symptoms met the definition of APS, which is considered to be one of the typical clinical features of NMOSD with great diagnostic value (4). This patient also developed LETM, which is also a characteristic of NMOSD. A previous study confirmed that when a longitudinally extensive spinal cord lesion extends to the area postrema and happens simultaneously with intractable nausea and vomiting, it is highly specific for AQP4 antibody-seropositive NMOSD (5). Thus, the typical presentation and neuroimaging of APS and LETM require more attention clinically.

We reviewed the previous literature and summarized the characteristics of the reported cases of NMOSD with seropositive AQP4 antibodies who presented with meningitis/meningomyelitis-like symptoms in the first attack (**Table 1**) (6–8). All patients had infection-like manifestations such as fever or headache in their early stages, along or subsequent with core symptoms of NMOSD. Brain MRI revealed that some of the cases have multiple T2 hyperintense lesions and meningeal enhancement. These symptoms were so similar to those of infectious meningomyelitis that left the diagnosis process in dilemma. However, previous reports showed that the lack of improvement in empiric antibiotic or anti-tuberculosis therapy favored the NMOSD attack (6, 8). Some patients were not correctly diagnosed at the first hospitalization but were further diagnosed when they relapsed during the follow-up (6). Most patients had relapses or disabilities. In comparison, our patient presented not only infectious meningomyelitis-like symptoms, which were similar to cases discussed above, but also APS in the earlier stage. Thanks to the timely AQP4 antibody test, the diagnosis successfully locked down on NMOSD, and the treatments focusing on NMOSD proved to be effective for our patient. Although meningitis/meningomyelitis-like phenotype is rare, it should have an attached importance, since delayed diagnosis and treatment may result in a poor prognosis.

**Table 1:** Clinical, MRI, and CSF characteristics of AQP4 antibody-seropositive NMOSD patients with meningitis/meningomyelitis-like symptoms at first attack.

Case report	Age, years/sex	Initial symptoms	Other symptoms during the courses of disease	Meningeal irritation	MRI		CSF		
					T2WI	Contrast enhancement	Cell count (/µl)	Protein level (mg/dl)	Other details
1. Wang et al. (6)	40/M	Headache	Fever, consciousness change	Kernig’s sign and nuchal rigidity	T2WI hyperintense in the right periventricular regions, corpus callosum, hypothalamus, cerebral peduncle, midbrain, and upper pons	Irregular parenchymal and meningeal gadolinium enhancement	606 (95% L)	285	310 mmH_2_O, 30.24 mg/dl glucose, normal chloride
2. Wang et al. (6)	38/F	Headache	Fever, APS, symptomatic narcolepsy, apathy	Kernig’s sign and nuchal rigidity	T2WI hyperintense lesions around the third ventricle, in the corpus callosum, bilateral periventricular parenchyma, temporal lobes, thalamus, cerebellum, and cerebellar peduncle	Irregular parenchymal, meningeal and bilateral ependymal gadolinium enhancement	625 (34.9% N)	102	200 mmH_2_O, normal glucose and chloride
3. Benedetti et al. (7)	45/F	Paresthesia	Fever, headache, ON, consciousness change, hyponatremia	Neck rigidity	T2WI hyperintense in the frontal lobes, optic tracts, optic chiasm, midbrain, anteroinferior region of basal ganglia, internal capsule, and hypothalamus, with swelling of the cerebral parenchyma	No enhancement	Pleocytosis (80 lymphocytes/mm^3^)	200	Absent OB
4. Shi et al. (8)	28/F	Fever, headache, acute myelitis	–	NA	NA	Cerebral meninge, spinal meninge enhancement	280 (33% N)	368	220 mmH_2_O, 12.06 mg/dl glucose, 113 mmol/l chloride
5. Shi et al. (8)	34/F	Fever, headache, acute myelitis	–	NA	NA	Spinal cord, spinal meninge enhancement	1,200 (60% N)	215.15	160 mmH_2_O, 32.4 mg/dl glucose, 121.4 mmol/l chloride
6. Shi et al. (8)	29/F	Fever, headache, acute myelitis	ON	NA	NA	Spinal cord, spinal meninge enhancement	1,131 (83% N)	158.67	220 mmH_2_O, 39.6 mg/dl glucose, 122.4 mmol/l chloride
7. (Index)	41/M	APS	Fever, headache, acute myelitis	Neck rigidity	T2WI hyperintensities from C2 to T8	No enhancement	1,250 (97% L)	864.4	180 mmH_2_O, normal glucose and chloride

APS, area postrema syndrome; AQP4, aquaporin-4; CSF, cerebrospinal fluid; F, female; L, lymphocyte; M, male; MRI, magnetic resonance imaging; N, neutrophil; NA, not available; NMOSD, neuromyelitis optica spectrum disorder; ON, optic neuritis; T2WI, T2-weighted imaging; WBC, white blood cell.

Furthermore, in our case, the progressive elevation of the CSF WBC count and the protein level was accompanied by the exacerbation of symptoms. In previous studies, CSF pleocytosis is present in around 50%–65% of samples (9, 10). A study including 211 AQP4 antibody-positive samples found that marked pleocytosis (CSF WBC ≥100/μl) was observed only in 6%, while protein levels exceeding 100 mg/dl were observed in about 29%, and CSF protein levels were correlated with the length of the spinal cord lesions indicated by a study (10). Previous research suggests CSF leukocyte counts as an inflammation indicator and protein concentrations as a blood–brain barrier (BBB) disruption indicator (11). In our case, the patient suffered progressively ascending CSF leukocyte counts and protein levels, which respectively indicate inflammation and the BBB disruption, and could further explain the progress severity of the patient’s symptoms. Our case suggested that the progressive growth in the CSF leukocyte count and the protein level cannot completely rule out the diagnosis of NMOSD.

The clinical symptoms of our patient improved with immunotherapy alone, also supporting the diagnosis of NMOSD. Unfortunately, the short-term therapeutic effect of this case was not satisfactory. The delayed diagnosis and treatment might be the critical factors. Previous studies found that tetraparesis and long spinal cord lesions were associated with long-term disability (12, 13). Since our case presented with these poor prognostic factors, follow-up is required to define the long-term prognosis.

In conclusion, we report a meningomyelitis-like case with distinct CSF findings that expands the phenotypic spectrum of NMOSD. To our knowledge, this is the first report that showed progressive elevation of the CSF WBC count and the protein level along with the aggravation of clinical symptoms. It may also lead to delayed diagnosis or even misdiagnosis. Although atypical symptoms exist, APS and LETM are still highly suggestive of the diagnosis of NMOSD. Testing for AQP4 antibody in this scenario is recommended, since it helps definite diagnosis and initiating therapy of this treatable disease in the early stage.

## Data Availability Statement

The original contributions presented in the study are included in the article/supplementary material. Further inquiries can be directed to the corresponding author.

## Ethics Statement

Written informed consent was obtained from the individual for the publication of any potentially identifiable images or data included in this article.

## Author Contributions

Y-XZ and T-YZ contributed to the concept and design of the study. All authors contributed to the acquisition and analysis of the data. Y-XZ and M-TC contributed to drafting the initial article. T-YZ contributed to revising the article for intellectual content. All authors read and approved the final version before submission.

## Conflict of Interest

The authors declare that the research was conducted in the absence of any commercial or financial relationships that could be construed as a potential conflict of interest.

## Publisher’s Note

All claims expressed in this article are solely those of the authors and do not necessarily represent those of their affiliated organizations, or those of the publisher, the editors and the reviewers. Any product that may be evaluated in this article, or claim that may be made by its manufacturer, is not guaranteed or endorsed by the publisher.
